# Development of Serum Marker Models to Increase Diagnostic Accuracy of Advanced Fibrosis in Nonalcoholic Fatty Liver Disease: The New LINKI Algorithm Compared with Established Algorithms

**DOI:** 10.1371/journal.pone.0167776

**Published:** 2016-12-09

**Authors:** Byron Lykiardopoulos, Hannes Hagström, Mats Fredrikson, Simone Ignatova, Per Stål, Rolf Hultcrantz, Mattias Ekstedt, Stergios Kechagias

**Affiliations:** 1 Department of Medical and Health Sciences, Linköping University, Linköping, Sweden; 2 Department of Medicine, Huddinge, Karolinska Institutet, Stockholm, Sweden; 3 Department of Clinical and Experimental Medicine, Linköping University, Linköping, Sweden; Medizinische Fakultat der RWTH Aachen, GERMANY

## Abstract

**Background and Aim:**

Detection of advanced fibrosis (F3-F4) in nonalcoholic fatty liver disease (NAFLD) is important for ascertaining prognosis. Serum markers have been proposed as alternatives to biopsy. We attempted to develop a novel algorithm for detection of advanced fibrosis based on a more efficient combination of serological markers and to compare this with established algorithms.

**Methods:**

We included 158 patients with biopsy-proven NAFLD. Of these, 38 had advanced fibrosis. The following fibrosis algorithms were calculated: NAFLD fibrosis score, BARD, NIKEI, NASH-CRN regression score, APRI, FIB-4, King´s score, GUCI, Lok index, Forns score, and ELF. Study population was randomly divided in a training and a validation group. A multiple logistic regression analysis using bootstrapping methods was applied to the training group. Among many variables analyzed age, fasting glucose, hyaluronic acid and AST were included, and a model (LINKI-1) for predicting advanced fibrosis was created. Moreover, these variables were combined with platelet count in a mathematical way exaggerating the opposing effects, and alternative models (LINKI-2) were also created. Models were compared using area under the receiver operator characteristic curves (AUROC).

**Results:**

Of established algorithms FIB-4 and King´s score had the best diagnostic accuracy with AUROCs 0.84 and 0.83, respectively. Higher accuracy was achieved with the novel LINKI algorithms. AUROCs in the total cohort for LINKI-1 was 0.91 and for LINKI-2 models 0.89.

**Conclusion:**

The LINKI algorithms for detection of advanced fibrosis in NAFLD showed better accuracy than established algorithms and should be validated in further studies including larger cohorts.

## Introduction

Nonalcoholic fatty liver disease (NAFLD) is the most prevalent liver disease in the Western world and a common reason for clinical evaluation due to elevated liver function tests [1]. The histopathological features of NAFLD include a wide spectrum of changes, ranging from simple steatosis to steatohepatitis and cirrhosis with risk of developing hepatocellular carcinoma [1]. Moreover, NAFLD has been established as a risk factor for cardiovascular morbidity and it is associated with an increased risk of metabolic disease, including diabetes [2].

Several follow-up studies have demonstrated increased mortality among patients with NAFLD. The main reason for this is attributed to excess mortality from cardiovascular diseases, but liver-related mortality is also greatly overrepresented [3, 4]. There is no consensus on which NAFLD patients that need to be monitored for early detection of future complications. However, hepatic fibrosis, particularly bridging fibrosis (stage 3) or cirrhosis (stage 4), seems to be the histological parameter that best predicts future risk of complications [5, 6]. Moreover, identification of NAFLD patients with cirrhosis is critical because screening for hepatocellular carcinoma and gastroesophageal varices is mandatory in these patients.

Liver biopsy is the clinical reference standard for assessing the stage of fibrosis but the method has well documented problems with sampling and interpretation variability as well as procedure related complications [7]. Liver biopsy is also expensive and difficult to access especially for general practitioners who encounter the majority of NAFLD patients. The limitations of liver biopsy have led to development of a variety of serum markers for identifying patients who are at risk for clinically significant hepatic fibrosis. The most common approach to assess the stage of fibrosis by serological means consists of routine biochemical and/or hematological tests. These are indirect serum markers and are based on the evaluation of common functional alterations in the liver, alterations that do not necessarily reflect extracellular matrix turnover and/or fibrogenic cell changes. A better understanding of the pathophysiology of liver fibrosis has prompted investigators to use more refined markers to identify different fibrosis stages. These, so called direct serum markers, are intended to detect extracellular matrix turnover and/or fibrogenic cell changes. Markers may be used alone or combined with other direct or indirect markers to form panels.

Several algorithms including a combination of indirect markers have been developed in NAFLD patients (BARD [8], NIKEI [9], NAFLD fibrosis score [10], NASH-CRN regression score [11]) as well as in patients with chronic hepatitis C virus (HCV) infection (GUCI [12], APRI [13], FIB-4 [14], King´s score [15], Forns score [16], Lok index [17]) (S1 Table). It is unclear whether the algorithms that were developed in NAFLD patients provide a better diagnostic accuracy. The Enhanced Liver Fibrosis (ELF) test is an example of a panel of direct markers, which highlight matrix turnover and consists of tissue inhibitor of matrix metalloproteinase 1 (TIMP 1), hyaluronic acid (HA), and aminoterminal peptide of pro-collagen III (P3NP) developed for a variety of liver disorders [18]. Although the ELF panel has been reported to have good diagnostic accuracy in NAFLD patients, the addition of indirect markers augments its diagnostic performance [19]. Other investigators have reported that one direct marker (HA) in combination with several indirect markers provides the best estimation of area of fibrosis measured with quantitative image analysis [11].

The objective of this study was to assess and compare the accuracy of non-invasive fibrosis algorithms to distinguish advanced fibrosis in NAFLD. Algorithms originally developed in NAFLD patients as well as in other liver diseases were compared. Moreover, we aimed at developing a new non-invasive model, (Linköping University-Karolinska Institute; LINKI) for predicting advanced fibrosis in NAFLD and to compare its diagnostic accuracy with well-established fibrosis algorithms.

## Patient and Methods

### Patients and data collection

We included 158 prospectively recruited patients between 2003 and 2013 from two tertiary centers, Linköping University Hospital (118 patients) and Karolinska University Hospital (40 patients). Patients had been referred from primary health care centers for evaluation of persistently (> 6 months) elevated serum alanine aminotransferase (ALT) and/or aspartate aminotransferase (AST). They underwent physical examination, ultrasonography of the liver, laboratory investigation, and liver biopsy as part of the clinical work-up. Medical history was scrutinized as well as information regarding alcohol consumption. Blood pressure, waist circumference, body weight, and height were measured. Subjects had blood drawn for routine clinical biochemical analyses at the local laboratories. These analyses included complete blood count and analysis of prothrombin time, transferrin, iron, transferrin saturation, ferritin, ALT, AST, alkaline phosphatase (ALP), gamma glutamyl transferase (GGT), bilirubin, carbohydrate deficient transferrin, fasting plasma glucose, total cholesterol, high-density lipoprotein, low-density lipoprotein, triglycerides, hepatitis B surface antigen, anti-HCV antibodies, antinuclear antibodies, smooth muscle antibodies, mitochondrial antibodies, albumin, α_1_-antitrypsin, immunoglobulins, and ceruloplasmin. Moreover, serum samples were obtained and stored at -80°C. These were subsequently thawed and analyzed for levels of TIMP-1, HA, and P3NP at an independent reference laboratory (Siemens Healthcare Diagnostics AB, Upplands Väsby, Sweden).

Included patients had been diagnosed with NAFLD, i.e. presence of hepatic steatosis at histopathological evaluation without evidence of alcohol consumption >140 g/week, any other concomitant liver disease, or medication associated with fatty infiltration of the liver.

### Liver biopsy and histopathological evaluation

A percutaneous liver biopsy was performed within 2 months after initial evaluation using a 16 gauge (1.6 mm) needle. The median (range) biopsy length was 20 mm (12–32 mm). Liver biopsies were assessed by two liver pathologists, one at each center, who were blinded for all clinical and laboratory patient data. Biopsies were graded and staged according to the Kleiner classification [20]. Fibrosis was staged on a 5-point scale: stage 0 = no fibrosis, stage 1 = zone 3 perisinusoidal/perivenular fibrosis, stage 2 = zone 3 and periportal fibrosis, stage 3 = septal/bridging fibrosis, stage 4 = cirrhosis. Advanced fibrosis was defined as stage 3 or 4. The degree of steatosis was graded 0–3. Grades 0–3 correspond to fat deposition in < 5%, 5–33%, 34–66%, and > 66% of the hepatocytes, respectively. Lobular inflammation was graded 0–3. Grades 0–3 correspond to none, < 2 foci, 2–4 foci, and > 4 foci per 200x field, respectively. Hepatocellular ballooning was graded 0–2. Grades 0–2 correspond to none, few balloon cells, and many cells/prominent ballooning, respectively. The NAFLD Activity Score (NAS) was calculated as the unweighted sum of the scores for steatosis (0–3), lobular inflammation (0–3), and hepatocellular ballooning (0–2).

### Non-invasive fibrosis algorithms

All relevant primary patient-level data are shown in S2 Table. The scores for 11 previously published non-invasive fibrosis algorithms were calculated according to the formulas provided in S1 Table.

### Statistical analysis

Statistical analyses were performed using SPSS (version 21; SPSS, Inc., Chicago, IL) unless otherwise specified. The Shapiro-Wilk test was used to test for normal distribution. Median (range) was calculated for continuous variables, frequencies for categorical variables. Continuous variables were compared using the Student’s *t* test or the Mann–Whitney *U* test when appropriate. The χ^2^ test or Fischer’s exact test were used to compare categorical variables. A two-sided p-value <0.05 was considered statistically significant if not specified otherwise. A Bonferroni correction of p-values was performed when multiple comparisons were made. Forward multiple logistic regression analysis was performed using bootstrapping, to identify the independent predictors of fibrosis with greater precision, and a new multivariate model (fibrosis score) was constructed.

Receiver-operating characteristics (ROC) curves of the tested scores were constructed for each subpopulation examined and the area under the ROC curve (AUROC) was calculated to assess the overall diagnostic accuracy of serum fibrosis algorithms and to identify optimal cut-offs. Bootstrap methods were applied with analysis of 1000 samples of the same size as the subsample with replacement and STATA (version 14; StataCorp LP, College Station, TX, USA) was used to calculate AUROCs and 95% confidence intervals (CI) of AUROCs. The sensitivity, specificity, positive predictive value (PPV), and negative predictive value (NPV) were calculated according to standard methods.

### Model building

#### First step

Patients from both centers were pooled, and 2/3 were then randomly assigned to the training group (n = 97) for model building and the remaining to the validation group (n = 61). Univariate statistics were performed to look for differences between the training and validation groups and to compare patients with (F3–F4) and without (F0–F2) advanced fibrosis. All variables with significant differences between the two fibrosis groups (AST, albumin, platelet count, glucose, prothrombin time, HA, P3NP, TIMP 1, age, presence of diabetes) (Table 1) along with other variables used in previously published fibrosis scores were included in a multivariate logistic regression analysis to identify those predicting the presence or absence of advanced fibrosis. Variables with p <0.05 in the regression analysis were used to construct a new model to predict advanced fibrosis (LINKI-1). Men were slightly overrepresented in the validation group but otherwise the two groups did not differ significantly (Table 2). Bootstrapping was then applied to confirm that the same predictors would be identified through the repeated sampling (with replacement) from the training group followed by a forward logistic regression analysis in each subsample (1000 subsamples of the same size as the subsample, with replacement, in our study) (Table 3). This procedure proved that the right predictors were identified from the beginning and that the model was stable.

**Table 1:** Demographic and laboratory characteristics of cohort. Values are median (range) or n (%).

Variable	Total cohort (n = 158)	No/mild fibrosis (n = 120)	Advanced fibrosis (n = 38)	*p* no/mild vs. advanced fibrosis
Age (yrs)	60 (19–83)	59 (19–83)	62 (49–80)	0.01*
Male (n;%)	117 (74%)	95 (79%)	22 (57%)	0.02*
Diabetes	82 (52%)	54 (45%)	28 (74%)	0.018*
BMI (kg/m^2^)	28.7 (20.2–49.5)	28.0 (20.2–49.5)	29.4 (23.4–44.1)	0.12
Overweight	76 (48%)	50 (42%)	18 (47%)	0.37
Obese	62 (39%)	39 (47%)	16 (42%)	0.35
AST (U/L)	38 (10–153)	34 (10–108)	53 (21–153)	<0.001*^#^
ALT (U/L)	58 (17–198)	55 (17–198)	60 (18–162)	0.29
AST/ALT ratio	0.7 (0.3–1.9)	0.7 (0.3–1.6)	0.9 (0.4–1.9)	<0.001*^#^
GGT (U/L)	63 (12–504)	55 (12–504)	96 (22–492)	0.07*
ALP (U/L)	100 (30–470)	82 (30–470)	112 (34–323)	0.29
Bilirubin (mg/dL)	11 (3–48)	11 (5–48)	12 (3–42)	0.13
Albumin (g/dL)	4.1 (2.9–4.9)	4.1 (3.1–4.9)	3.9 (2.9–4.9)	<0.001*^#^
Platelet count (x10^9^/L)	221 (85–476)	230 (85–476)	195 (88–336)	0.011*
Prothrombin (INR)	1.0 (0.8–1.3)	1.0 (0.8–1.2)	1.0 (0.9–1.3)	0.02*
Ferritin (μg/L)	192 (5.0–1804)	186 (14–1804)	223 (5–723)	0.51
Transferrin (g/L)	2.3 (1.6–5.2)	2.2 (1.6–5.2)	2.4 (1.6–4.1)	0.87
Transferrin saturation	0.27 (0.09–0.55)	0.33 (0.09–0.55)	0.38 (0.16–0.35)	0.58
Glucose (mmol/L)	7.2 (3.8–18)	6.0 (3.8–15.7)	7.9 (5.3–18)	<0.001*^#^
Cholesterol (mg/dL)	187 (93–320)	195 (93–323)	169 (113–320)	0.037*
HDL (mg/dL)	43 (12–174)	46 (12–174)	35 (31–120)	0.08
Triglycerides (mg/dL)	125 (43–507)	123 (43–481)	165 (85–507)	0.07
HA (μg/L)	45 (6–795)	38.5 (6–795)	104 (12–356)	<0.001*^#^
P3NP (μg/L)	7.7 (2.0–28)	7.0 (2.0–28)	10.2 (3.9–23)	<0.001*^#^
TIMP 1 (μg/L)	248 (70–735)	241 (70–591)	302 (132–735)	<0.001*^#^
Steatosis (grade)	2 (1–3)	2 (1–3)	2 (1–3)	0.15
Lobular inflammation (grade)	0 (0–3)	0 (0–3)	1 (0–3)	<0.001*^#^
Ballooning (grade)	0 (0–2)	0 (0–2)	0.5 (0–2)	<0.001*^#^
NAS	2 (1–7)	2 (1–7)	3.5 (1–7)	0.007*

Abbreviations: BMI, body mass index; AST, aspartate aminotransferase; ALT, alanine aminotransferase; GGT, gamma glutamyltransferase; ALP, alkaline phosphatase; INR, international normalized ratio; HDL, high density lipoprotein; HA, hyaluronic acid; P3NP, aminoterminal peptide of pro-collagen III; TIMP-1, tissue inhibitor of metalloproteinase 1; NAS, NAFLD Activity Score.

*p <0.05

^#^Significant differences after using the Bonferroni correction for 30 comparisons. This resulted in adjustment of the p-value denoting significant differences from 0.05 to 0.0017.

Variables age, albumin, platelet count, and cholesterol were normally distributed. For these variables comparisons were performed with Student’s *t* test. The remaining continuous variables were compared with the Mann–Whitney *U* test. The χ^2^ test was used to compare categorical variables.

**Table 2:** Demographic and laboratory characteristics of total cohort, training group and validation group. Values are median (range) or n (%).

Variable	Total cohort (n = 158)	Training group (n = 97)	Validation group (n = 61)	*p* training vs. validation group
Age (yrs)	60 (19–83)	62 (19–83)	57 (26–82)	0.89
Men (n; %)	117 (74%)	69 (71%)	48(78%)	0.02*^¶^
Diabetes (%)	82 (52%)	58(60%)	24 (41%)	0.89
BMI (kg/m^2^)	28.7 (20.2–49.5)	28.2 (20.4–49.5)	29 (20.2–42.5)	0.87
Overweight	76 (48%)	47 (48%)	29 (47%)	0.87
Obese	62 (39%)	39 (40%)	23 (38%)	0.85
AST (U/L)	38 (10–153)	41 (10–120)	14 (21–153)	0.62
ALT(U/L)	58 (17–198)	57 (17–198)	60 (21–181)	0.48
AST/ALT ratio	0.7 (0.3–1.9)	0.8 (0.3–1.9)	0.7 (0.3–1.9)	0.77
GGT (U/L)	63 (12–504)	58 (12–492)	66 (17–504)	0.42
ALP (U/L)	100 (30–470)	94 (30–470)	112 (30–341)	0.87
Bilirubin (mg/dL)	11 (3–48)	11 (3–48)	11 (5–36)	0.29
Albumin (g/dL)	4.1 (2.9–4.9)	4.0 (3.4–4.9)	4.1 (2.9–4.9)	0.6
Platelet count (x10^9^/L)	221 (85–476)	219 (85–476)	223 (133–369)	0.89
Prothrombin (INR)	1.0 (0.8–1.3)	1.0 (0.9–1.2)	1.0 (0.8–1.3)	0.96
Ferritin (μg/L)	192 (5–1804)	178 (5–1120)	217 (16–1804)	0.14
Transferrin (g/L)	2.3 (1.6–5.2)	2.3 (1.6–5.2)	2.3 (1.6–3.5)	0.28
Transferrin saturation	0.27 (0.09–0.55)	0.32 (0.09–0.55)	0.34 (0.16–0.36)	0.32
Glucose (mmol/L)	7.16 (3.8–18)	6.6 (4.0–18)	5.8 (3.8–18)	0.19
Cholesterol (mg/dL)	187 (93–320)	183 (93–324)	181 (94–320)	0.42
HDL (mg/dL)	43 (12–174)	44 (12–120)	199 (27–174)	0.76
Triglycerides (mg/dL)	125 (43–507)	124 (43–481)	142 (47–507)	0.50
HA (μg/L)	45 (6–795)	49 (6–356)	42.5 (7–795)	0.76
P3NP (μg/L)	7.7 (2.0–28)	8.0 (2.0–28)	6.7 (2.0–24)	0.58
TIMP 1 (μg/L)	248 (70–735)	253 (70–526)	243 (76–735)	0.88
Steatosis (grade)	2 (1–3)	2 (1–3)	2 (1–3)	0.78
Lobular inflammation (grade)	0 (0–3)	0 (0–3)	1 (0–3)	0.88
Ballooning (grade)	0 (0–2)	0 (0–2)	0 (0–2)	0.25
NAS	2 (1–7)	2 (1–7)	2 (1–7)	0.90
Fibrosis (stage)	1 (0–4)	1 (0–4)	1 (0–4)	0.89

Abbreviations: BMI, body mass index; AST, aspartate aminotransferase; ALT, alanine aminotransferase; GGT, gamma glutamyltransferase; ALP, alkaline phosphatase; INR, international normalized ratio; HDL, high density lipoprotein; HA, hyaluronic acid; P3NP, aminoterminal peptide of pro-collagen III; TIMP-1, tissue inhibitor of metalloproteinase 1; NAS, NAFLD Activity Score.

*p <0.05

^¶^Non-significant difference after using the Bonferroni correction for 30 comparisons. This resulted in adjustment of the p-value denoting significant differences from 0.05 to 0.0017.

Variables age, albumin, platelet count, and cholesterol were normally distributed. For these variables comparisons were performed with Student’s *t* test. The remaining continuous variables were compared with the Mann–Whitney *U* test. The χ^2^ test was used to compare categorical variables.

**Table 3:** Logistic regression results (training group) based on 1000 bootstrap samples.

Variable	Coefficient	*p*	95% CI Lower limit	95% CI Upper limit
HA (μg/L)	0.019	0.02*	0.005	0.03
AST (U/L)	0.0888	0.02*	0.025	0.112
Glucose (mmol/L)	0.34	0.02*	0.064	0.544
Age (yrs)	0.066	0.01*	0.029	0.158
Constant	-24.136			

Abbreviations: HA, hyaluronic acid; AST, aspartate aminotransferase, CI, confidence interval.

*p <0.05

Those variables selected were further tested in numerous mathematical combinations in order to derive three additional indexes (LINKI-2a, LINKI-2b, LINKI-2c) that exaggerated the effects of the opposing predictors (positive and negative) in accordance with what has previously been performed by other authors when developing FIB-4 and King´s score [14, 15].

#### Second step, validation group

The novel LINKI models were applied in the validation group and comparisons were conducted with those of the previously published fibrosis models that performed best in the training group (AUROC >0.80). AUROCs were calculated and bootstrap methods were applied for a second time to assess their confidence intervals.

#### Third step, total study cohort

In the final step, all patients were pooled and ROC curves were constructed for all fibrosis models included in the study, AUROCs were calculated and bootstrap methods were applied for a third time. For each of the four LINKI models two cut-off points were selected in order to provide an NPV and a PPV near 90%. NPV, PPV, sensitivity and specificity were also determined for established fibrosis models using previously published cut-off levels in NAFLD patients. The same principles were applied for selection of optimal NAFLD specific cut-offs for King´s score and GUCI, which have not been validated in NAFLD patients previously.

### Ethical considerations

The study was approved by the Regional Ethical Review Board in Linköping and the Regional Ethical Review Board in Stockholm, Sweden (www.epn.se/en/start). Written informed consent was obtained from the participants.

## Results

### Characteristics of study cohort

The demographic and laboratory characteristics of all included patients (n = 158), of whom 122 (74%) were male, are shown in Table 1. Median age was 60 years and the majority (78%) was overweight or obese and 52% had diabetes. No complications occurred due to liver biopsy. Distribution of various fibrosis stages was as follows: stage 0, n = 49 (31%); stage 1, n = 39 (25%); stage 2, n = 32 (20%); stage 3, n = 20 (13%); stage 4, n = 18 (11%).

### Training group

By applying bootstrap logistic regression in the training group four variables remained significant: age, HA, AST, and glucose. Using these variables we constructed a new algorithm to predict advanced fibrosis.

LINKI-1 = (Age (yrs) x 0.066) + (AST (U/L) x 0.0888) + (glucose (mmol/L) x 0.34) + (HA (μg/L) x 0.019)– 24.136 (Table 4).

**Table 4:** Novel non-invasive fibrosis algorithms.

**LINKI-1:** HA (μg/L) AST (U/L), glucose (mmol/L), age (yrs)	(age x 0.066)+(AST x 0.0888)+(glucose x 0.34)+(HA x 0.019)-24.136
**LINKI-2a:** HA (μg/L), AST (U/L), glucose (mmol/L), age (yrs), Platelet count (x10^9^/L)	HA x AST^2^ x age x (glucose)/(platelet count)
**LINKI-2b:** HA (μg/L), AST (U/L), glucose (mmol/L), age (yrs), platelet count (x10^9^/L)	HA x AST x age x (glucose)^2^/(platelet count)
**LINKI-2c:** HA (μg/L), AST (U/L), glucose (mmol/L), age (yrs), platelet count (x10^9^/L)	HA x AST x age x (glucose)/(√platelet count)

Abbreviations: LINKI, Linköping University-Karolinska Institute; AST, aspartate aminotransferase; HA, hyaluronic acid

The AUROC for LINKI-1 in the training group was 0.92 (95% CI 0.86–0.97), (Table 5).

**Table 5:** Area under the receiver-operating characteristics curves (95% CI) of the best performing fibrosis algorithms and APRI.

Fibrosis algorithm	Training group (n = 97)	Validation group (n = 61)	Total cohort (n = 158)
LINKI-1	0.92 (0.86–0.97)	0.93 (0.86–0.99)	0.91 (0.87–0.96)
LINKI-2a	0.87 (0.80–0.95)	0.92 (0.85–0.99)	0.89 (0.84–0.94)
LINKI-2b	0.88 (0.80–0.96)	0.90 (0.80–0.99)	0.89 (0.83–0.95)
LINKI-2c	0.88 (0.77–0.95)	0.93 (0.86–0.99)	0.89 (0.84–0.95)
FIB-4	0.81 (0.72–0.90)	0.93 (0.85–1.0)	0.84 (0.79–0.92)
NASH-CRN regression score	0.84 (0.75–0.92)	0.76 (0.58–0.93)	0.81 (0.73–0.89)
King´s score	0.81 (0.71–0.91)	0.94 (0.87–1.0)	0.83 (0.78–0.93)
ELF	0.77 (0.65–0.90)	0.85 (0.73–0.97)	0.78 (0.70–0.89)
NAFLD fibrosis score	0.76 (0.65–0.88)	0.84 (0.71–0.96)	0.79 (0.72–0.89)
APRI	0.73 (0.60–0.84)	0.88 (0.78–0.99)	0.78 (0.72–0.89)

Mathematical combinations that exaggerated the effects of the opposing factors selected above were used in order to generate three other algorithms (LINKI-2a, LINKI-2b, LINKI-2c). Key component in this procedure is the use of products and ratios instead of sums of factors. The formulas for all LINKI algorithms are shown in Table 4. The AUROCs of LINKI-2a, LINKI-2b, and LINKI-2c in the training group were 0.87 (95% CI 0.80–0.95), 0.88 (95% CI 0.80–0.96), and 0.88 (95% CI 0.77–0.95), respectively (Table 5).

The AUROCs of previously published serum fibrosis algorithms were also calculated in the training group. All LINKI algorithms compared favorable to the established algorithms (Table 5).

Algorithms with lower AUROCs (NAFLD fibrosis score 0.76, Forns score 0.75, GUCI 0.74, APRI 0.73, BARD 0.72, Lok index 0.72, and NIKEI 0.72) were omitted from further comparisons in the validation group.

### Validation group

AUROCs for LINKI models as well as those of the previously published serum fibrosis models with the best performance in the training group were also calculated in the validation group. Most notably was that King´s score and FIB-4 had substantially higher AUROCs in the validation group compared with the training group. AUROCs for LINKI algorithms were similar in the validation and training groups (Table 5).

### Total study cohort

**Fig 1:**
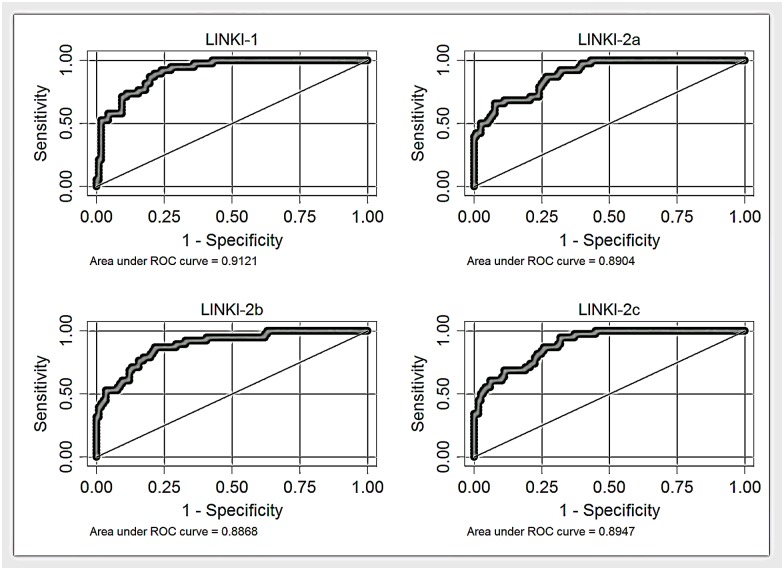
Receiver-operating characteristic (ROC) curves (total study cohort) for LINKI algorithms.

**Fig 2:**
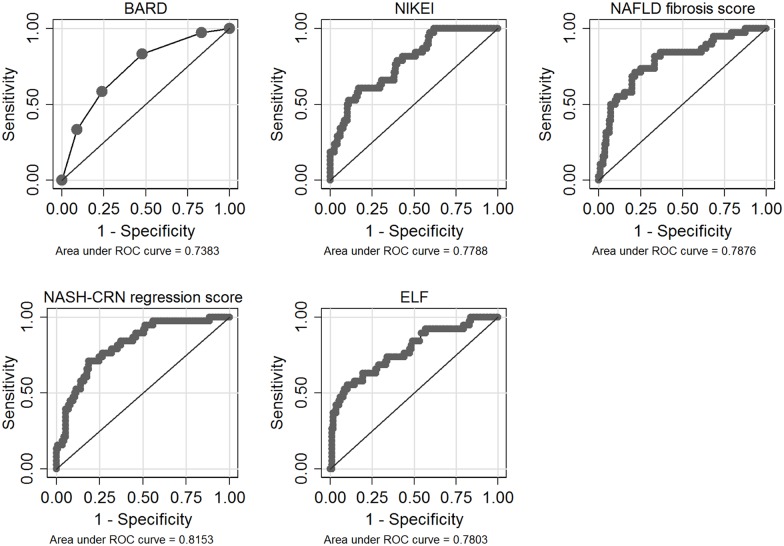
Receiver-operating characteristic (ROC) curves (total study cohort) for ELF and previously published algorithms developed in NAFLD patients.

**Fig 3:**
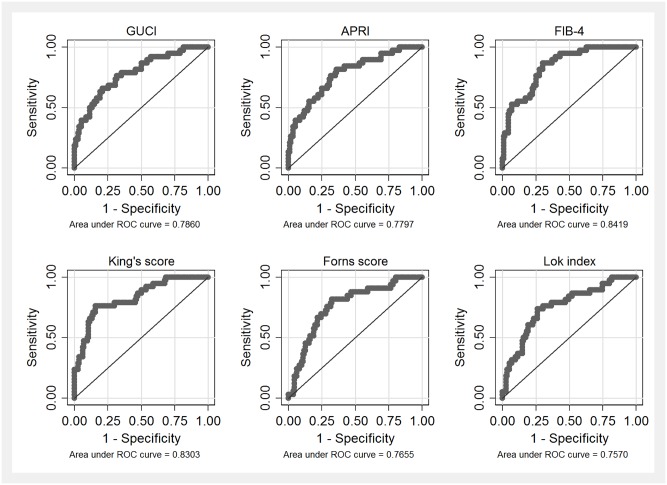
Receiver-operating characteristic (ROC) curves (total study cohort) for previously published algorithms developed in patients with hepatitis C virus infection.

ROC curves for all fibrosis algorithms in the total study cohort are shown in Figs 1–3. AUROCs of the best performing fibrosis algorithms in the total study cohort are presented in Table 5. The differences in AUROCs between LINKI models, King´s score, NASH-CRN regression score, ELF, and FIB-4 were not significant with the exception of LINKI-1 vs ELF.

Algorithms with poorer performance (AUROC <0.80) in the total study cohort were NAFLD fibrosis score 0.79 (95% CI 0.72–0.89), APRI 0.78 (95% CI 0.72–0.89), ELF 0.78 (95% CI 0.70–0.89), NIKEI 0.78 (95% CI 0.68–0.86), Forns score 0.77 (95% CI 0.68–0.85), GUCI 0.76 (95% CI 0.71–0.90), Lok index 0.76 (95% CI 0.66–0.85), and BARD 0.74 (95% CI 0.65–0.84), respectively.

Due to the anticipated effect of glucose-lowering agents on fasting glucose in patients with diabetes receiving pharmacological treatment, a modified analysis of the diagnostic performance of LINKI models was also performed. All glucose values were replaced with either a fixed value of 5.5 mmol/L (median glucose value in non-diabetics) in patients without diabetes or with a fixed value of 8.6 mmol/L (median glucose value in diabetics) in patients with diabetes. This resulted in a somewhat reduced AUROC for LINKI-1 (0.90 vs. 0.91), LINKI-2a (0.88 vs. 0.89), LINKI-2b (0.88 vs. 0.89), and LINKI-2c (0.88 vs. 0.89). However, despite this correction, LINKI algorithms still performed better than established fibrosis algorithms.

The performance of the different LINKI algorithms in discriminating absence (F0) vs. any (F1-F4) fibrosis stage or F0-F1 vs. F2-F4 was inferior when compared with their ability to discriminate between F0-F2 vs. F3-F4 or between F0-F3 vs. F4 (Table 6).

**Table 6:** Performance of LINKI fibrosis algorithms in discriminating various fibrosis stages. Values denote area under the receiver-operating characteristics (ROC) curves (95% CI).

Fibrosis algorithm	F0 vs. F1-F4	F0-F1 vs. F2-F4	F0-F2 vs. F3-F4	F0-F3 vs. F4
LINKI-1	0.79 (0.71–0.86)	0.81 (0.74–0.88)	0.91 (0.87–0.96)	0.91 (0.85–0.96)
LINKI-2a	0.79 (0.71–0.86)	0.79 (0.72–0.86)	0.89 (0.84–0.94)	0.92 (0.86–0.98)
LINKI-2b	0.78 (0.70–0.85)	0.78 (0.71–0.86)	0.89 (0.83–0.95)	0.93 (0.87–0.99)
LINKI-2c	0.79 (0.72–0.87)	0.79 (0.72–0.86)	0.89 (0.84–0.95)	0.92 (0.86–0.98)

By applying a lower cut-off point of -11 and a higher cut-off point of -10, NPV and PPV for LINKI-1 were 0.89 (105/119) and 0.83 (20/24), respectively. With these cut-off points 15 patients (12.6%) were classified as indeterminate. Similar or slightly higher NPVs and PPVs could be achieved with LINKI-2 algorithms, however at the expense of more patients being classified as indeterminate. A lower cut-off of -13.8 resulted in an excellent NPV for LINKI-1, 0.98 (59/60), however leading to classification of 74 patients (46%) into the indeterminate group (Table 7).

**Table 7:** Sensitivities, specificities, negative predictive values and positive predictive values in the total cohort for novel and previously published fibrosis algorithms.

Fibrosis algorithm	Lower cut-off	NPV	Upper cut-off	PPV	Ind	Mis	Se	Spe
LINKI-1	-13.8	0.98 (59/60)	-10	0.83 (20/24)	74 (46%)	4 (6%)	53%	49%
LINKI-1	-11	0.89 (105/119)	-10	0.83 (20/24)	15 (12.6%)	17 (12%)	53%	88%
LINKI-2a	125 000	0.94 (75/80)	800 000	0.86 (18/21)	57 (36%)	8 (8%)	47%	63%
LINKI-2b	40 000	0.9 (93/103)	130 000	0.88 (16/18)	37 (23%)	12 (10%)	42%	78%
LINKI-2c	2 500 000	0.91 (86/95)	10 000 000	0.86 (19/22)	41 (26%)	12 (10%)	50%	72%
FIB-4	1.3	0.945 (70/74)	2.67	0.72 (13/18)	66 (42%)	9 (10%)	34%	58%
King´s score	11	0.9 (82/92)	26	0.82 (9/11)	55 (35%)	12 (12%)	24%	68%
NAFLD-fibrosis score	-1.455	0.87 (46/53)	0.676	0.66 (14/21)	84 (53%)	14 (19%)	44%	37%
ELF	7.7	0.9 (82/92)	9.8	1(7/7)	59 (37%)	10 (6%)	18%	68%
GUCI	0.2	1 (19/19)	1	0.79 (11/14)	125 (79%)	3 (11%)	37%	16%
Forns score	4.2	0.88 (30/34)	6.9	0.48 (16/33)	91 (58%)	21 (33%)	42%	25%
NIKEI	0.0535	0.83 (94/113)	0.2294	0.61 (14/23)	23 (15%)	28 (20%)	35%	75%
APRI	0.5	0.85 (86/101)	1.5	1 (5/5)	52 (33%)	15 (14%)	13%	74%
Lok index	0.2	0.85 (68/80)	0.5	0.67 (8/12)	66 (42%)	16 (17%)	21%	57%
BARD	<2	0.89 (59/66)	>2	0.36 (30/84)	8 (5%)	61 (41%)	79%	49%

Abbreviations: NPV, negative predictive value; PPV, positive predictive value; Ind, indeterminate; Mis, misclassification (of non-indeterminate); Se, sensitivity; Spe, specificity.

Diagnostic performance of established non-invasive fibrosis algorithms were calculated using previously published cut-offs in NAFLD patients with the exception of King´s score, Forns score, Lok index, and GUCI, which have not been validated in NAFLD patients previously and therefore published cut-offs for patients with HCV infection were used. An NPV of 0.9 was almost universally achieved but at the expense of substantially more patients being classified into the indeterminate group compared with the LINKI algorithms (Table 7).

Stepwise combination with previously published fibrosis algorithms either before or after application of LINKI models did not increase diagnostic accuracy.

## Discussion

Established serum fibrosis algorithms composed of indirect or direct serum fibrosis markers had a moderate diagnostic accuracy when applied independently. We therefore assessed if a combination of indirect and direct markers would perform better in discriminating advanced fibrosis and found that the well-known indirect fibrosis markers, age, glucose, and AST combined with the direct fibrosis marker HA to form the LINKI-1 algorithm had the best diagnostic accuracy. Stability of the novel algorithm was internally confirmed by bootstrapped multiple regression analysis where the same factors were selected repeatedly most of the times and therefore included into the final model, LINKI-1. A cut-off of -13.8 resulted in a very high NPV of 0.98, i.e. correctly identifying in essence all patients without advanced fibrosis. Values > -10 resulted in a PPV of 0.83 for advanced fibrosis. Using these thresholds 6% of patients were misclassified and 46% were classified into the indeterminate category. Only King´s score resulted in less patients classified into the indeterminate category. With a more liberal lower cut-off of -11, that might be more useful for assessment of NAFLD patients in primary healthcare, an NPV of 0.89 was achieved and only 12.6% of patients were classified as indeterminate (Table 7, Fig 2).

Low platelet count, even if not selected by multiple regression analysis in the LINKI-1, was significantly negatively correlated with fibrosis stage as shown in previous studies [21]. LINKI-2a, LINKI-2b, and LINKI-2c were developed as ratios and not as simple sum-scores in order to balance the weight effect of positive predictive factors used in the LINKI-1 with a negative fibrosis stage predictor. These types of scores may have a better generalizability in populations with different characteristics in accordance with what other authors attempted when complex quotients such as FIB-4 and King´s score were developed [14, 15]. Interestingly, with the exception of the LINKI models, FIB-4 and King´s score had the best diagnostic accuracy in our cohort. We chose to present three alternative algorithms rather than one since the developing process was not a product of a single statistical analysis such as multiple regression analysis but rather a selection of three mathematical combinations between many that were initially tested. Although LINKI-2a, LINKI-2b, and LINKI-2c were shown to have marginally inferior diagnostic performance than LINKI-1, future studies will determine if they are more stable than LINKI-1 and which one has the best diagnostic performance.

It should be noted that the difference between the AUROCs of the various fibrosis scores did not reach statistical significance with the exception of LINKI-1 vs. ELF and LINKI-1 vs. APRI. The cohort in the present study was rather small and future studies using larger cohorts are required to determine which algorithms have significantly better diagnostic performance in NAFLD patients.

Of previously published algorithms assessed in this study NAFLD fibrosis score, BARD, NIKEI, and NASH-CRN regression score have been developed in NAFLD patients. The NAFLD fibrosis score is one of the most validated and has been implemented by Dyson et al. in a proposed algorithm for managing patients with suspected NAFLD in primary care [22]. A meta-analysis reported AUROCs for NAFLD fibrosis score between 0.80 and 0.93 [23] and in a recently published study, in which 452 NAFLD patients were included, the AUROC was only 0.73 [24]. Similar results were obtained in our evaluation with an AUROC of 0.79 (95% CI 0.72–0.89). Disappointingly, as many as 53% of NAFLD patients were classified into the indeterminate group at an NPV of 0.87. Thus, neither we nor Boursier et al. [24] were able to confirm that the NAFLD fibrosis score is clinically useful to rule out advanced fibrosis in NAFLD. BARD [8] is simple to calculate in clinical practice and has previously been reported to perform well in order to exclude advanced fibrosis in NAFLD [25]. In our cohort we found an AUROC of 0.74 (95% CI 0.65–0.84), a fairly good NPV (0.89) but at the expense of a low PPV (0.36) and 41% of patients being misclassified. Our results are in accordance with those of Boursier et al. [24], who showed moderate diagnostic performance for BARD (AUROC 0.70). NIKEI is a newly developed NAFLD specific fibrosis score [9]. In its original publication, a very high diagnostic accuracy was reported with an AUROC of 0.97 and an NPV of 0.99. In our study, AUROC and NPV of NIKEI were considerably lower, 0.78 (95% CI 0.68–0.86) and 0.83, respectively, thus not differing significantly from other moderately performing scores.

The ELF score, which composes of three direct serum fibrosis markers, has shown an AUROC of 0.90 for discriminating advanced fibrosis in NAFLD with an NPV of 0.94. Combining the direct markers with simple markers such as age, BMI, presence of diabetes, AST/ALT-ratio, albumin, and platelet count improved AUROC to 0.98 [19]. The performance of ELF in our cohort was disappointing with a much lower AUROC of 0.78 (95% CI 0.70–0.89). However, of the three components of ELF, HA was used in the LINKI algorithms. HA has been previously shown to correlate well with advanced fibrosis [21] and our results is also in accordance with a previous study [11] which reported that HA in combination with several indirect markers provides the best estimation of area of fibrosis measured with quantitative image analysis.

Of the algorithms originally developed in patients with HCV infection, GUCI [12], APRI [13], Forns score [16], and Lok index [17], performed less well than other algorithms. GUCI, Forns score, and Lok index have previously not been evaluated in NAFLD patients. Studies of the diagnostic performance of APRI in NAFLD have shown conflicting results with AUROCs ranging from 0.75 [24] to 0.85 [26].

King´s score has previously not been evaluated in NAFLD patients [15]. We found a fairly good AUROC of 0.83 (95% CI 0.78–0.93) and with previously reported cut-offs in patients with HCV infection PPV and NPV were 0.82 and 0.90, respectively with 33% of patients being classified into the indeterminate group. These results are promising and must be confirmed in other studies before this score can be recommended for clinical use in NAFLD. FIB-4 was originally developed from a cohort of 832 HIV/HCV co-infected patients [14]. This algorithm has previously been evaluated in NAFLD with AUROCs of 0.80 [27] and 0.78 [24], and NPVs of 0.90 [27] and 0.82 [24]. In our study, the performance of FIB-4 was even better with an AUROC of 0.84 (95% CI 0.79–0.92) and an NPV of 0.94, implying the best diagnostic accuracy of previously published serum fibrosis algorithms. It should however be emphasized that with established cut-offs, 42% of patients were classified into the indeterminate group.

Liver stiffness measurement by transient elastography (FibroScan) was initially reported to perform very well (AUROC 0.93) for the non-invasive diagnosis of advanced fibrosis in NAFLD [28]. However, a recent study [24] showed a moderate diagnostic accuracy (AUROC 0.83), and according to current guidelines [29] LSM requires further validation in NAFLD. In our cohort, LSM was performed in only 62 out of 158 patients. Thus, we are not able to perform a thorough comparison between LSM and included serum fibrosis algorithms. Interestingly, also within this limited cohort of 62 patients, AUROC was highest for LINKI-1 (0.95) and the remaining LINKI algorithms had higher AUROCs than the corresponding AUROC for LSM (0.86).

A limitation of studies, including ours, assessing serum fibrosis markers is that liver biopsy is used as reference standard for evaluation of hepatic fibrosis. Important limitations of liver biopsy are its known sampling variability, the subjective nature of fibrosis staging and the high inter-observer variability [7]. The limitations of liver biopsy probably impair the potential of fibrosis algorithms to reach the state of perfect surrogate fibrosis markers [30].

Our study was undertaken in two tertiary centers where NAFLD patients were mainly referred by general practitioners for evaluation of abnormal liver function tests. A selection bias for referral and for decision which patients should undergo liver biopsy cannot be ruled out and constitutes a second limitation of the present study. Prevalence of different fibrosis stages is known to affect the observed AUROCs. In patients with HCV infection adjusted-DANA (regression formula for standardizing AUROCs estimated from populations which differ in distribution of fibrosis stages) has been developed to overcome this confounder and increase the comparability of fibrosis algorithms. So far no similar concept exists for NAFLD [31].

Compared with a well-designed study of NAFLD patients in primary care [32] the prevalence of diabetes in our cohort was higher (52.5% vs 38.5%) while body mass index (BMI) was lower (28.7 vs 31.5 kg/m^2^) and age slightly higher (58.8 vs 58 years). The prevalence of advanced fibrosis cannot be directly compared because liver biopsies were not performed in the other study. However, less patients (34% vs. 57%) had a low NAFLD fibrosis score in our study while more patients (13% vs. 8%) had a high NAFLD fibrosis score indicating that advanced fibrosis might be more prevalent in our cohort. As a consequence, NPVs of LINKI algorithms may be even higher in the general NAFLD population and fewer patients may be classified into the indeterminate group. In a previous study [4], in which all patients referred for evaluation of elevated liver function tests underwent liver biopsy, our group showed that 129 subjects out of 212 had NAFLD. Eight NAFLD patients (6.2%) had advanced fibrosis (F3-F4). This result is in accordance with a study from the US [33], in which 9 out of 129 subjects with NAFLD had significant fibrosis (F2-F4). In the present study, the prevalence of advanced fibrosis in NAFLD patients was 24%, indicating that patients that underwent liver biopsy were selected by the hepatologists because of high probability of having advanced fibrosis. Assuming that the prevalence of advanced fibrosis in subjects with NAFLD of the general population is 6%, the use of LINKI-1 with a lower cut-off point of -11 would provide a very high NPV (0.96) but at the expense of a lower PPV (0.53). LINKI-1 could thus be potentially useful in primary care to exclude advanced fibrosis in NAFLD patients.

The sensitivity and specificity of LINKI algorithms were rather low. The use of two cut-offs gives priority to the predictive values before sensitivity and specificity in algorithms with limited diagnostic accuracy. However, high predictive value, particularly NPV, may still be of clinical utility in the context of NAFLD since most subjects will not have advanced fibrosis.

Specific factors that may limit the usefulness of LINKI are that conditions unrelated to hepatic fibrosis can confound the results. HA can be affected by many conditions including inflammatory diseases, renal failure, and may also be prone to significant intraindividual variation [34–38]. Moreover, quantification of HA is not widely accessible but a broader introduction of the method may be justified if other studies confirm our results.

In conclusion, the LINKI fibrosis algorithms are promising and may provide superior diagnostic accuracy compared to previously reported fibrosis algorithms in NAFLD. They may be useful in primary care to ‘‘rule out” NAFLD patients with advanced fibrosis needing referral for monitoring of liver-related complications. In secondary care settings they may be useful to ‘‘rule in” NAFLD patients with advanced fibrosis thereby reducing the need to perform liver biopsy. However, our results lack external validation and need to be evaluated in future studies.

## Supporting Information

S1 Table Previously published non-invasive fibrosis algorithms assessed in the study. (DOCX)

S2 Table Primary patient-level data. (XLSX)

## Abbreviations

ALP: alkaline phosphatase
ALT: alanine aminotransferase
AST: aspartate aminotransferase
AUROC: area under the receiver operator characteristic curve
BMI: body mass index
CI: confidence intervals
ELF: Enhanced Liver Fibrosis
GGT: gamma glutamyl transferase
HA: hyaluronic acid
HCV: hepatitis C virus
INR: international normalized ratio
LINKI: Linköping University-Karolinska Institute
LSM: liver stiffness measurement
NAFLD: nonalcoholic fatty liver disease
NPV: negative predictive value
P3NP: aminoterminal peptide of pro-collagen III
PPV: positive predictive value
ROC: receiver-operating characteristics
TIMP 1: tissue inhibitor of matrix metalloproteinase 1
