# Supplementary material for: Development of Serum Marker Models to Increase Diagnostic Accuracy of Advanced Fibrosis in Nonalcoholic Fatty Liver Disease: The New LINKI Algorithm Compared with Established Algorithms
Source: PLoS One. 2016 Dec 9;11(12):e0167776. doi: 10.1371/journal.pone.0167776 (PMC5147971; doi:10.1371/journal.pone.0167776)
Supplement: S1 Table — (DOCX) [file pone.0167776.s001.docx]

|  | Calculation formula | Reported lower cut-off | Reported higher cut-off |
| --- | --- | --- | --- |
| NAFLD fibrosis score^10^ | -1.675 + 0.037 x age (yrs) + 0.094 x BMI (kg/m^2^) +1.13 x impaired fasting glucose/diabetes (yes = 1, no = 0) + 0.99 x AST/ALT ratio – 0.013 x platelet count (x10^9^/L) – 0.66 x albumin (g/dL). | Absence of F3-F4: < -1.455 | Presence of F3-F4: > 0.676 |
| APRI^13^ | (AST (U/L)/(AST upper limit of normal))/(platelet count (×10^9^/L) × 100) | Absence of F3-F4: < 0.50 | Presence of F3-F4: > 1.50 |
| FIB-4^14^ | (age (yrs) x AST (U/L)) / ((platelet count (×10^9^/L)) x (ALT(U/L))^1/2^) | Absence of F3-F4: < 1.30 | Presence of F3-F4: > 2.67 |
| BARD^8^ | BMI > 28 kg/m^2^ = 1, AST/ALT > 0.8 = 2, presence of diabetes = 1 | Absence of F3-F4: < 2 | Presence of F3-F4: > 2 |
| NIKEI^9^ | -24.214 + 0.225 x age (yrs) + 0.056 x AST (U/L) + 5.044 x AST/ALT ratio + 3.631 x bilirubin (mg/dL) | Absence of F3-F4: < 0.0535 | Presence of F3-F4: > 0.2294 |
| NASH-CRN regression score^11^ | 0.0539 x body weight (kg) + 10.5166 x glucose (mmol/L) + 10.0695 x AST (U/L)-0.0189 x ALT (U/L) - 0.0594 x prothrombin index (%) -3.6323 |  |  |
| King´s score^15^ | ((age (yrs) x AST (U/L) x prothrombin (INR)) / (platelet count (10^9^/L)) | Absence of F2-F4: < 11 | Presence of F2-F4: > 26 |
| GUCI^12^ | AST (µkat/L) / (AST upper limit of normal) (µkat/L) x prothrombin (INR) x 100 / platelet count (× 10^9^/L) | Absence of F3-F4: < 0.2 | Presence of F3-F4: > 1 |
| ELF^16^ | 2.2781 + 0.851 x ln [HA] (µg/L) +10.751 x ln [P3NP] (µg/L) +10.934 x ln [TIMP 1] (µg/L) | Absence of F3-F4: < 7.7 | Presence of F3-F4: > 9.8 |
| Lok index^17^ | log odds = - 5.56 – 0.0089 x platelet count (10^3^/mm^3^) + 1.26 x (AST/ALT) + 5.27 x INR;  Lok = [exp (log odds)]/[1 + exp (log odds)] | Absence of F3-F4:  < 0.2 | Presence of F3-F4:  > 0.5 |
| Forns score^16^ | 7.811 - 3.131 x ln [platelet count (10^9^  /L)] + 0.781 x ln [GGT(U/L)] + 3.467 x ln [age (yrs)] – 0.014 [cholesterol (mg/dL)] | Absence of F3-F4:  < 4.2 | Presence of F3-F4:  > 6.9 |
